# Nano-Phase and SiC–Si Spherical Microstructure in SiC/Al-50Si Composites Solidified under High Pressure

**DOI:** 10.3390/ma16124283

**Published:** 2023-06-09

**Authors:** Rong Zhang, Chunming Zou, Zunjie Wei, Hongwei Wang, Chuang Liu

**Affiliations:** 1 School of Materials Science and Engineering, Fujian University of Technology, Fuzhou 350118, China; 2 School of Materials Science and Engineering, Harbin Institute of Technology, Harbin 150001, China; zouchunming1977@163.com (C.Z.); wanghw@hit.edu.cn (H.W.);

**Keywords:** high pressure, nano-phase precipitation, SiC–Si spherical microstructure, SiC/Al-50Si composites, properties

## Abstract

The formation of coarse primary Si is the main scientific challenge faced in the preparation of high-Si Al matrix composites. The SiC/Al-50Si composites are prepared by high pressure solidification, which allows the primary Si to form a SiC–Si spherical microstructure with SiC, while the solubility of Si in Al is increased by high pressure to reduce the proportion of primary Si, thus enhancing the strength of the composites. The results show that the high melt viscosity under high pressure makes the SiC particles almost “fixed” in situ. The SEM analysis shows that the presence of SiC in the growth front of the primary Si will hinder its continued growth and eventually form SiC–Si spherical microstructure. Through aging treatment, a large number of dispersed nanoscale Si phases are precipitated in the α-Al supersaturated solid solution. The TEM analysis shows that a semi-coherent interface is formed between the α-Al matrix and the nanoscale Si precipitates. The three-point bending tests shows that the bending strength of the aged SiC/Al-50Si composites prepared at 3 GPa is 387.6 MPa, which is 18.6% higher than that of the unaged composites.

## 1. Introduction

Silicon carbide-reinforced aluminum-silicon (SiC/Al-Si) matrix composites have the advantages of low density, high specific strength, high dimensional stability, and high thermal conductivity and have been widely used in aerospace, automotive, electronics, and other fields [1,2,3]. The distribution of reinforcement in metal matrix composites has a significant effect on the strength, plasticity, and crack initiation of the composites [4,5,6]. Segurado et al. [7] obtained composites with a non-uniform distribution of reinforcements, and their tensile strength was increased by 60% compared with uniformly distributed composites. Al matrix composites are reinforced by the continuous distribution of Al_2_O_3_ short fibers, resulting in greater strength and toughness than conventional composites with uniformly distributed reinforcements [8,9,10,11]. The addition of SiC particles to the Al-Si alloy results in a continuous SiC–Si network structure, which increases the elastic modulus of the composite [12]. In the above Al matrix composites with low Si content (<12 wt.%), the reinforcement formed a network structure with eutectic Si, resulting in the improvement in composite properties. In contrast, in Al matrix composites with high Si content (>40 wt.%), the effect of microstructure formed by continuous distribution of reinforcements and coarse primary Si on properties is relatively less studied.

High-pressure (HP) solidification has the advantages of improving microstructure uniformity, refining microstructure, and increasing solute solubility [13,14,15,16,17,18,19,20,21], and these characteristics are beneficial to improving the properties of SiC/Al-Si composites [22,23,24,25]. The Al-20Si alloy prepared at 3 GPa showed the microstructural characteristics of a hypoeutectic alloy; the pressure played a refining role on the eutectic Si; the hardness of the Al-Si eutectic phase was 144 HV; the tensile strength of the Al-20Si alloy was 365 MPa; and the elongation was 2.98% [22]. Compared with the specimens at 1 atm, the hardness, tensile strength, and elongation of the Al-20Si alloys prepared at 3 GPa were improved by 61%, 83%, and 413%, respectively. The 45 vol.% SiC/Al-20Si composites prepared at 3 GPa had high relative density, high thermal conductivity (166.8 W/m∙K), and a low thermal expansion coefficient (7.8 × 10^−6^ /K) [24,25]. The HP solidification method improves the relative density of the composites and significantly reduces the microporous defects in the composites, which is beneficial to the properties of the composites. The challenge in the practical application of materials prepared under HP is the instability after pressure release [14]. The new materials prepared by HP must be transformed from a sub-stable microstructure at HP to a stable microstructure at atmospheric pressure for better practical applications.

When the Si content is more than 20%, coarse primary Si is easily formed, which leads to stress concentration and reduces the strength of the composite [6]. The formation of coarse primary Si is the main scientific challenge faced in the preparation of high-silicon aluminum matrix composites. Pressure can change the distance between atoms and cause changes in crystal structures, so HP solidification has great potential in material preparation [14,15,16,17,18,19,20]. It is important to explore microstructure evolution and strengthening mechanisms under high pressure condition. The SiC/Al-50Si composites are prepared by HP solidification, which allows the primary Si to form a SiC–Si spherical microstructure with SiC, while the solubility of Si in Al is increased by high pressure to reduce the proportion of primary Si, thus enhancing the strength of the composites.

In this paper, the SiC–Si spherical microstructure and nano Si phase precipitation of SiC/Al-50Si composites were produced by HP solidification and aging treatment processes. The formation mechanism of the SiC–Si spherical microstructure and the solid solubility of Si in the Al matrix under HP were revealed, and the bending strength of the composites were investigated. The HP solidification method provides an effective way to enhance high-silicon aluminum matrix composites.

## 2. Experimental Procedures

**Figure 1:**
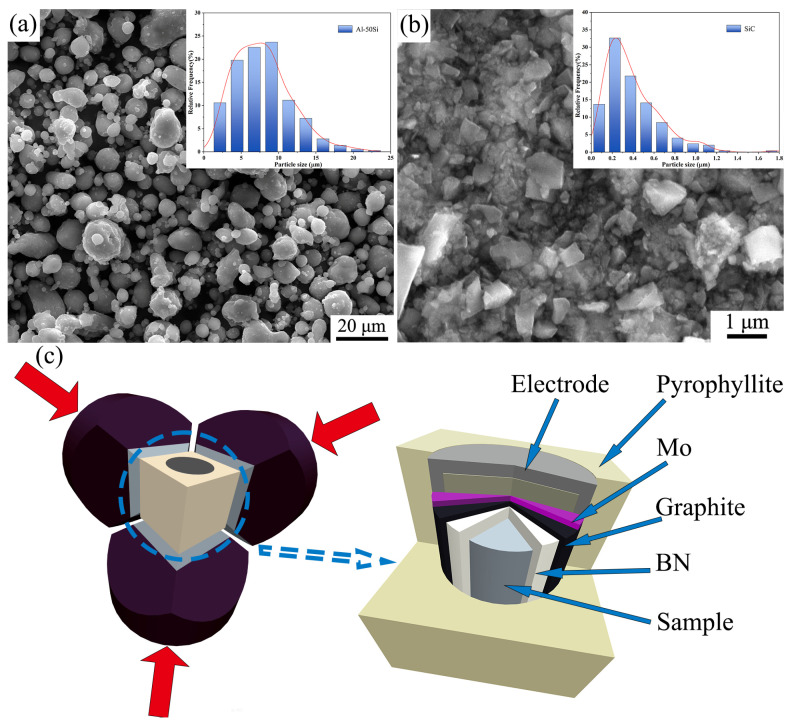
Raw materials and equipment for HP experiment. (**a**) Al-50Si alloy powders; (**b**) SiC particles; (**c**) cell assembly.

Al-50 wt.%Si alloy powders (purity 99.8 wt.%) and SiC particles (purity 99.7 wt.%) were used as raw materials. The morphology and size of the powders are shown in Figure 1a,b. The Al-50Si and SiC powders, in the 80:20 volume ratio, were dry mixed in a ball mill at a speed of 90 rpm for 6 h. The interior of the ball mill tank is tetrafluoroethylene, the balls are non-magnetic stainless steel, and the protective gas is argon. At a pressure of 300 MPa, the mixed powders were then compacted into cylinders of Φ20 × 18 mm. The SiC/Al-50Si composites were prepared using the HTDS-032F HP apparatus shown in Figure 1c. The pressure was calibrated by the phase transition of Bi (at 2.55 GPa) [26], and the temperature was determined by a B-type thermocouple. Pressure and temperature calibrations were conducted in a separate experiment using the same conditions [21]. The sample was first encapsulated in a covered boron nitride (BN) cylinder, then the BN cylinder was encapsulated in a covered graphite cylinder, and finally the graphite cylinder was encapsulated in a pyrophyllite cavity using molybdenum (Mo) sheets and electrode plugs. The HP experimental procedure was first pressurized to 1–3 GPa, then melted at 1323–1423 K, and finally cooled to room temperature at a cooling rate of 20 K/s. The samples prepared by HP were removed from the pyrophyllite and artificially aged at 433 K for 12 h.

The samples were ground with SiC paper and polished with a diamond solution. The samples underwent etching with 0.5 vol.% hydrofluoric acid solution for 10 s. To analyze the microstructure, an OLYMPUS optical microscope, a ZEISS-Merlin-Compact scanning electron microscope operating at 20 kV, and a FEI-Talos-F200X transmission electron microscope (TEM) operating at 200 kV were used. The phases were identified through Empyrean X-ray diffractometer (XRD). Three-point bending tests were performed on the resulting samples utilizing an Instron 3382 testing machine with sample sizes of 19 × 3 × 2 mm and a bending speed of 0.3 mm/min. The densities of the composites were measured by Archimedes’ principle. All tests were repeated five times to obtain good reproducibility of data. Statistical measurements of nanoscale particles were obtained by the ImagePro Plus 6.

## 3. Results and Discussion

### 3.1. Microstructure

**Figure 2:**
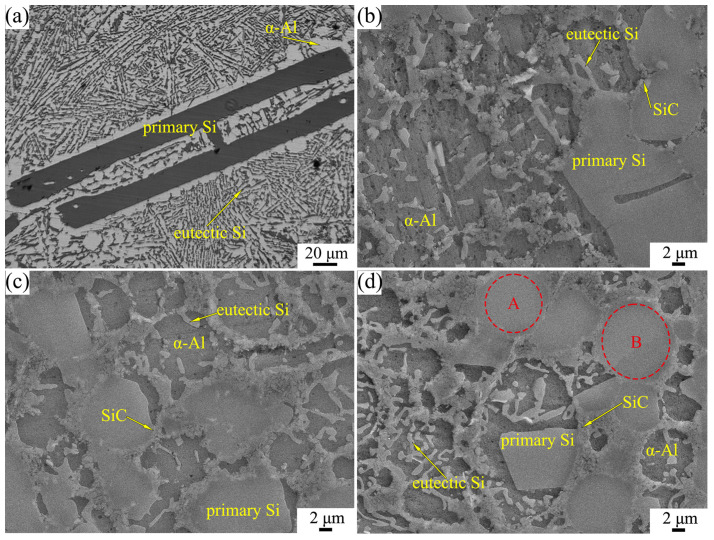
Microstructures of (**a**) Al-50Si alloys and (**b**–**d**) SiC/Al-50Si composites fabricated under HP: (**a**,**b**) 1 GPa; (**c**) 2 GPa; (**d**) 3 GPa.

Figure 2 shows the microstructure of the high-silicon aluminum materials prepared at HP. As shown in Figure 2a, the primary Si in the Al-50Si alloy at 1 GPa is large in size and mostly slate-like in morphology. As shown in Figure 2b–d, the Al-Si eutectic spacing decreases with increasing pressure, which is consistent with our previous results obtained in the Al-20Si alloy [27]. In the SiC/Al-50Si composites prepared by HP, the SiC particles are distributed in the matrix as a network, forming a SiC–Si spherical microstructure with the primary Si, which effectively changes the morphology of the primary Si, as shown in the dashed lines A and B in Figure 2d.

### 3.2. Formation Mechanism of SiC–Si Spherical Microstructure

The distribution of SiC particles in the Al-Si alloy melt under HP determines the solidification microstructure morphology of SiC/Al-50Si composites. According to the Arrhenius equation, the ratio of melt viscosity at HP to atmospheric pressure can be expressed as [28]:

(1) $$\frac{\eta_{P}}{\eta }=\exp\left(\frac{PV_{0}}{RT}\right)$$

where *η_P_* is the melt viscosity at HP, *η* is the melt viscosity at atmospheric pressure, *V*_0_ is the initial molar volume of the liquid, *T* is the temperature, *P* is the pressure, and *R* is the gas constant. For Al-50Si alloy, *V*_0_ = 1.12 × 10^−5^ m^3^/mol [29].

**Figure 3:**
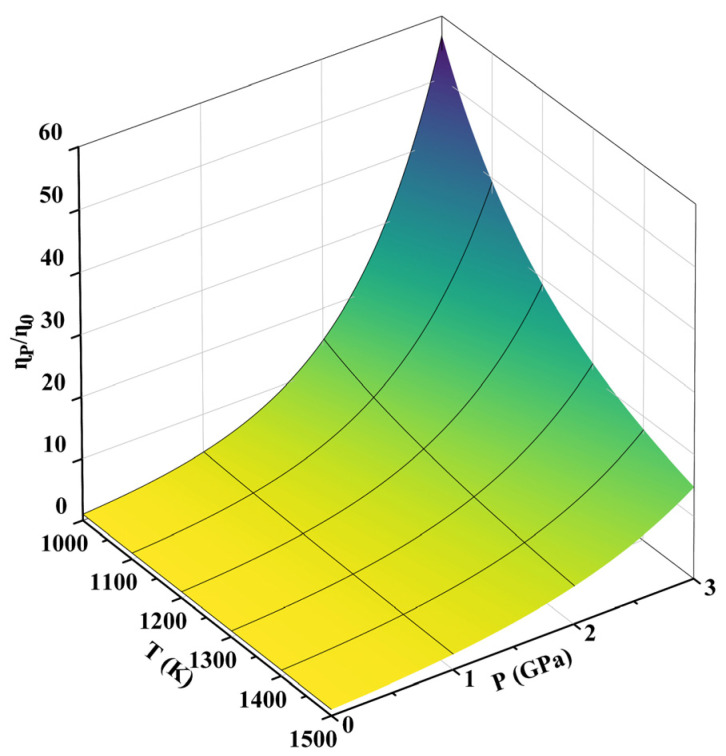
Effect of pressure and temperature on the melt viscosity of Al-50Si alloy.

The relationship between melt viscosity, pressure, and temperature in Al-50Si alloy can be obtained according to Equation (1), as shown in Figure 3. The increase in pressure from 1 atm to 3 GPa and the decrease in temperature from 1500 K to 1000 K both cause an increase in melt viscosity. The influence of both on the melt viscosity is close in the calculated pressure and temperature ranges. The viscosity of the Al-50Si alloy melt at 3 GPa is about 10 times higher than that at atmospheric pressure, which inhibits convection and the movement of SiC particles in the melt.

**Figure 4:**
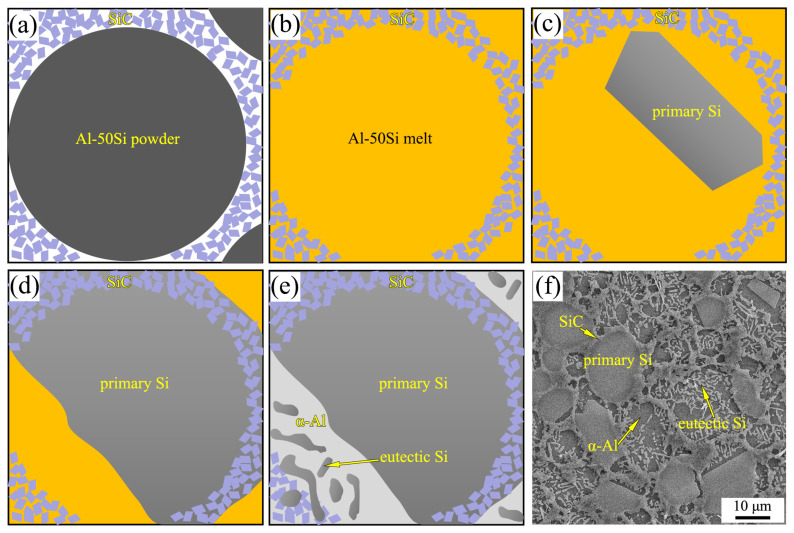
SiC–Si spherical microstructure in SiC/Al-50Si composites fabricated under HP: schematic illustration of (**a**) cold pressing, (**b**) melting, and (**c**–**e**) solidification process; (**f**) microstructure.

Figure 4 shows a schematic illustration of the formation process of SiC–Si spherical microstructure in SiC/Al-50Si composites under HP. Since the SiC particles are much smaller than the Al-50Si alloy particles, the SiC particles are evenly distributed in the voids of the Al-50Si alloy particles after powder mixing, so the SiC particles after cold pressing are evenly distributed around each matrix alloy particle, as shown in Figure 4a. When the Al-50Si alloy powders are melted under HP conditions, the SiC particles are almost “fixed” in situ due to the high viscosity of the melt, and the SiC particles basically maintain the initial network distribution, as shown in Figure 4b. The SiC particles are able to act as heterogeneous nucleation particles of the Si phase during HP solidification, allowing the Si phase to nucleate and grow on them. The boundary of the primary Si is relatively flat when its growth is not hindered by the network distribution of SiC particles, as shown in Figure 4c. When SiC particles are present at the front of the growing solid–liquid interface of the primary Si, it will hinder the continued growth of the primary Si, thus forming a SiC–Si spherical microstructure with the primary Si, as shown in Figure 4d. With the growth of primary Si, Al atoms are enriched in the front of the solid–liquid interface to form a high Al concentration zone, and the α-Al phase is attached to the primary Si to precipitate first, and then to form a lamellar Al-Si eutectic, as shown in Figure 4e. The final solidification microstructure formed is shown in Figure 4f.

The HP solidification method has the potential to prepare network-structured composites. Using the characteristics of high melt viscosity under HP, it is possible to prepare composites with a finer structure in the future.

### 3.3. Nano-Phase Precipitation

**Figure 5:**
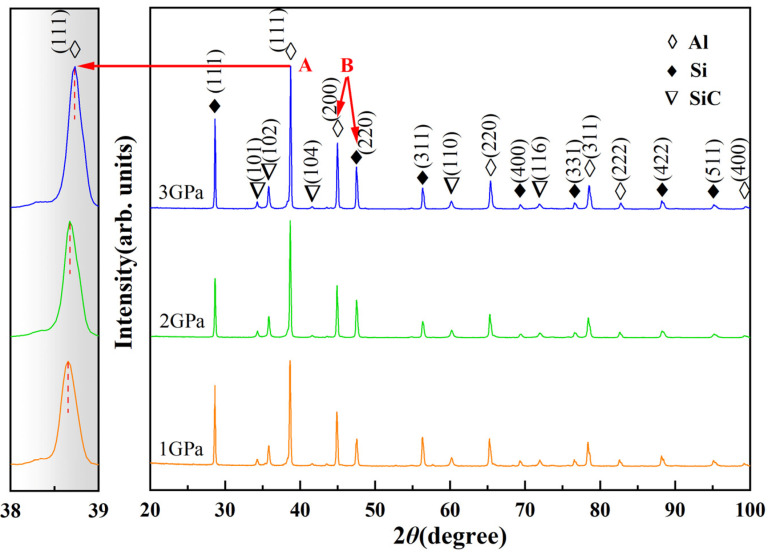
X-ray diffraction patterns of the SiC/Al-50Si composites fabricated under HP.

Figure 5 shows the XRD patterns of SiC/Al-50Si composites at different solidification pressures. According to the powder diffraction files (PDF) of Al(PDF#04-0487), Si(PDF#27-1402), and SiC(PDF#29-1131), the diffraction peaks of the Al, Si, and SiC phases are identified at 1–3 GPa. As the pressure increases, the angle of the (111) diffraction peak corresponding to α-Al becomes larger, as shown by the arrow at A in Figure 5. According to the Bragg equation:

(2) $$2d\sin\theta_{d}=\lambda$$

where *d* is the crystal plane spacing, *θ*_d_ is the diffraction angle, and *λ* is the wavelength.

According to Equation (2), *λ* is constant, and the diffraction angle *θ*_d_ of the α-Al phase becomes larger, causing a decrease in the crystal plane spacing *d*. Since the Si atomic radius (~1.17 Å) is smaller than the Al atomic radius (~1.43 Å), the increase in pressure causes an increase in the solid solubility of the Si in the α-Al matrix, which decreases the α-Al phase crystal plane spacing.

The solid solubility of Si in the α-Al matrix can be calculated based on a fitted relational equation based on experimental data [30]:

(3) $$a_{\mathrm{Al}}=0.40491-0.0174x_{\mathrm{Si}}$$

(4) $$a_{\mathrm{Al}}=0.40491-0.0174x_{\mathrm{Si}}-0.0144x_{\mathrm{Si}}^{2}$$

where *a*_Al_ is the lattice constant of Al and *x*_Si_ is the content of Si in the matrix.

The difference between Equations (3) and (4) is due to the different number of Si clusters present in the α-Al phase. The distribution of Si atoms in the α-Al matrix has a significant influence on its lattice parameters. If the Si atoms in the α-Al matrix are diffusely distributed, the reduction of the lattice parameters of the α-Al phase is smaller and close to the estimation result of Equation (3). If the Si atoms in the α-Al matrix are distributed in clusters, the reduction in the lattice parameters of the α-Al phase is larger and close to the estimation result of Equation (4). This may be related to the presence of Si-Si bonds in the Si clusters.

The lattice parameters of the α-Al matrix were calculated according to the diffraction peak angle of the α-Al phase (111) plane in Figure 5 and Equation (2), and the solid solubility of Si in the composites was estimated according to Equations (3) and (4), and the calculation results are shown in Table 1. As the pressure increases, the solid solubility of Si in the matrix increases from 2.5–2.6 at.% at 1 GPa to 8.1–8.7 at.% at 3 GPa. Since the TEM can only observe very thin regions, the observation of Si clusters does not allow us to fully determine their volume fraction. Therefore, the exact number of Si clusters cannot be determined, and only the solid solubility of Si in the α-Al phase can be roughly estimated.

Based on the lattice parameters of the Al phase, the solubility of Si in the Al-20Si alloys is 1.85, 5.27, and 6.73 at.% at 1, 2, and 3 GPa, respectively [23]. Compared to the Al-20Si alloys, the solubility of Si is slightly higher in the SiC/Al-50Si composites, indicating that the higher Si content in the matrix increases the solubility of Si under high pressure.

**Figure 6:**
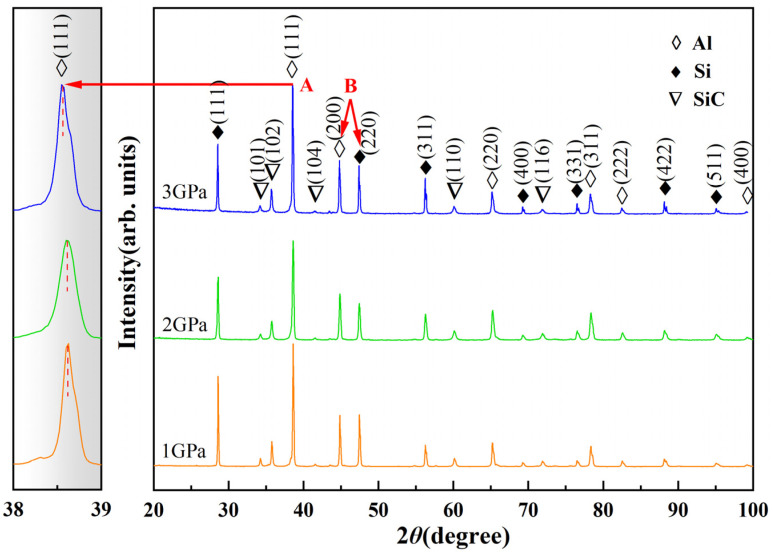
X-ray diffraction patterns of the aged SiC/Al-50Si composites fabricated under HP.

Figure 6 shows the XRD patterns of the aged SiC/Al-50Si composites at different solidification pressures. After aging treatment, the angles of the corresponding (111) diffraction peaks of α-Al at 1–3 GPa decrease to almost the same, as shown by the arrow at A in Figure 6. The relative intensities of the diffraction peaks of the Si phases are significantly higher after aging treatment, as shown by the arrows at B in Figure 5 and Figure 6. The arrows at A and B both indicate the precipitation of Si in the Al phase after aging treatment.

### 3.4. Mechanical Properties

**Figure 7:**
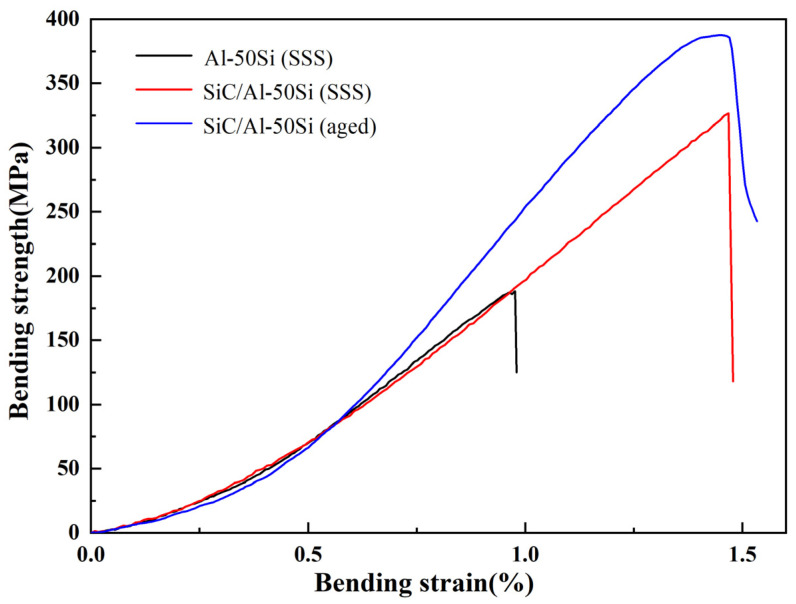
Bending stress–strain curves of materials prepared under 3 GPa.

According to our previous study, SiC/Al-20Si composites prepared at 3 GPa had the highest mechanical properties and density in the pressure range of 1–3 GPa [21]. The increasing pressure improves the density of the composites and significantly reduces the microporous, thereby enhancing the strength. In this study, the densities of the composites prepared at 1, 2, and 3 GPa pressures were 2.585 ± 0.0019, 2.5982 ± 0.002, and 2.614 ± 0.002 g/cm^3^, respectively. So, the materials prepared at 3 GPa were selected for bending tests in this study. The bending properties of the materials prepared at 3 GPa are shown in Figure 7. Before aging treatment, the Al matrix in Al-50Si alloy and SiC/Al-50Si composites is supersaturated solid solution (SSS). The bending strength of the SiC/Al-50Si composite prepared at 3 GPa is 326.7 MPa, which is 73.4% higher than that of the Al-50Si alloy. In addition, the bending strength of the aged SiC/Al-50Si composites is 387.6 MPa, which is 18.6% higher than that before aging treatment.

The fracture stress of primary Si can be expressed as [31]:

(5) $$\sigma_{f}=\frac{1}{Y}\sqrt{\frac{2E\gamma_{\mathrm{IC}}}{\pi C}}$$

where *Y* is the dimensionless correction factor, *E* is the Young’s modulus, *γ*_IC_ is the fracture surface energy, and *C* is the crack size.

**Figure 8:**
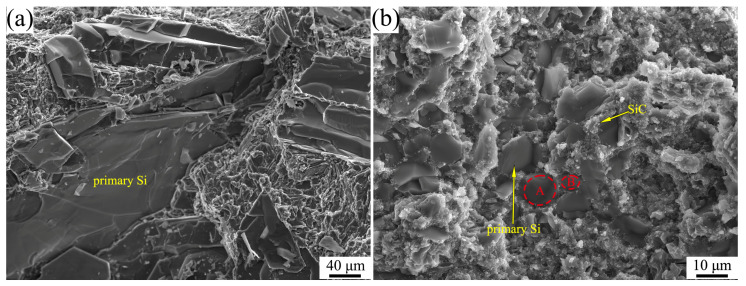
Bending fracture morphologies of materials prepared at 3 GPa. (**a**) Al-50Si; (**b**) SiC/Al-50Si.

Figure 8 shows the bending fracture morphologies of the materials prepared at 3 GPa. During the growth and coalescence of dimples, large plastic deformation takes place [32]. Therefore, the elongation of the Al-50Si is mainly attributed to the ductile fracture of the α-Al matrix, as shown in Figure 8a. According to Equation (5), the primary Si in Al-50Si alloy breaks along the cleavage plane, the crack propagation resistance and length are small, and the fracture surface energy is small, resulting in low bending strength and elongation of the alloy. The SiC–Si spherical microstructure in SiC/Al-50Si composites changes the primary Si from an overall brittle fracture to a multi-regional brittle fracture, which increases the crack propagation resistance and length, resulting in an increase in the fracture surface energy and thus an increase in the strength and elongation of the composite, as shown by the dashed lines A and B in Figure 8b. The bonding state between SiC particles and primary Si is fine, which can change the direction of crack propagation.

**Figure 9:**
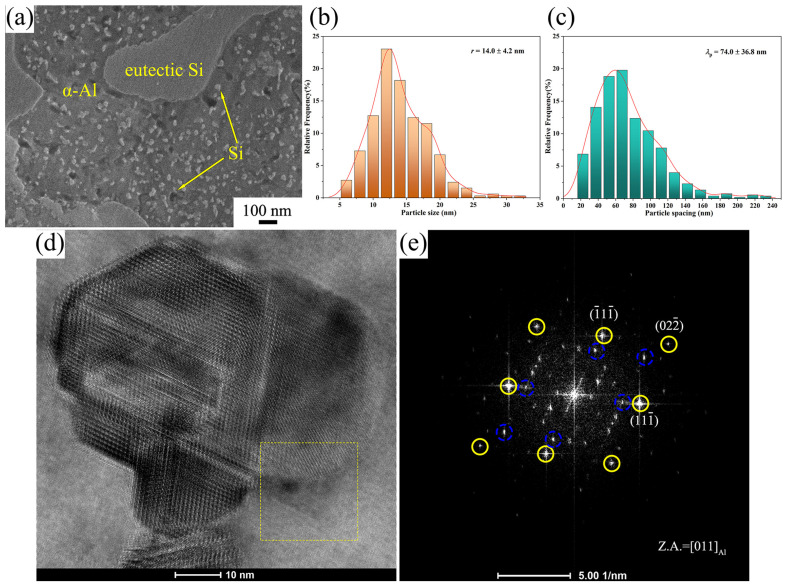
Microstructure of SiC/Al-50Si composites prepared at 3 GPa after aging treatment. (**a**) nanoscale Si phases; (**b**) particle size of nanoscale Si phases; (**c**) particle spacing of nanoscale Si phases; (**d**) high resolution TEM images; (**e**) fast Fourier transform pattern of the selected area in (**d**).

Figure 9 shows the microstructure of the aged SiC/Al-50Si composite prepared at 3 GPa. Through aging treatment, a large number of dispersed nanoscale Si phases are precipitated in the α-Al supersaturated solid solution, as shown in Figure 9a. As shown in the dashed box in Figure 9d, the boundary of the Si precipitated phase is approximately circular, and it can be presumed that the nucleation shape of the Si precipitated phase is approximately spherical. As shown in Figure 9e, the diffraction spot obtained by the fast Fourier transform consists of the Al matrix, the Si phase, and the double diffraction. The orientation of diamond-cubic-structured Si phases (blue dashed lines) is identical to that of the face-centered cubic-structured Al matrix (yellow solid lines). A semi-coherent interface is formed between the α-Al matrix and the nanoscale Si precipitates.

Unshearable nanoscale Si particles enhance the properties of the composites through the Orowan bypass mechanism. The calculation is based on the following Orowan equation [33]:

(6) $$\Delta \sigma_{\mathrm{Orowan}}=M\frac{0.4G_{m}b}{\pi \lambda_{p}}\frac{\ln(2\bar{r}/b)}{\sqrt{1-v}}$$

(7) $$\bar{r}=\sqrt{2/3}r$$

(8) $$\lambda_{p}=2\bar{r}\left(\sqrt{\frac{\pi }{4V_{p}}}-1\right)$$

where *M* is the average orientation factor, *G*_m_ is the shear modulus of the matrix, *b* is the Burgers vector of the matrix, *v* is the Poisson’s ratio, *r* is the average radius of the particles, $\bar{r}$ is the average radius of a circular cross section of spherical particles in a random plane, and *λ*_p_ is the average spacing between particles.

For the α-Al matrix, *M* = 3.06, *G*_m_ = 27.9 GPa, *b* = 0.286 nm, *v* = 0.33. The statistical measurements of the nanoscale Si phase particles were: *r* = 14.0 ± 4.2 nm, *λ*_p_ = 74.0 ± 36.8 nm, as shown in Figure 9b,c. According to the Orowan equation, Δσ_Orowan_ = 256.3 MPa. Since the precipitation strengthening caused by the precipitation of nanoscale Si particles acts only on the α-Al matrix, the volume fraction of the primary Si and SiC particles needs to be excluded. The volume fraction of the α-Al matrix was theoretically calculated to be about 37.1%, so the contribution of nanoscale Si phase particles in the α-Al matrix to the yield strength was Δσ_Orowan_ = 83.4 MPa.

Table 2 shows the bending strength of Al-50Si matrix alloy with different elements or reinforcements. The results show that the addition of 20 vol.% SiC can effectively improve the bending strength of the Al-50Si matrix alloy. This method can be combined with microalloying technology to further improve the strength of the Al-50Si matrix alloy.

## 4. Conclusions

In this paper, the SiC/Al-50Si composites were produced by HP solidification. The high viscosity of the melt at high pressure is used to obtain a SiC–Si spherical microstructure for material strengthening. The high solute solubility at high pressure is used to prepare a supersaturated solid solution, and the material is strengthened by the nano-phase precipitated by aging treatment. The conclusions are as follows:

(1) The high melt viscosity under HP makes the SiC particles almost “fixed” in situ. The presence of SiC in the growth front of the primary Si will hinder its continued growth and eventually form a SiC–Si spherical microstructure.
(2) The solid solubility of Si in the Al matrix was increased from 2.5–2.6 at.% at 1 GPa to 8.1–8.7 at.% at 3 GPa. Through aging treatment, a large number of dispersed nanoscale Si phases are precipitated in the α-Al supersaturated solid solution. A semi-coherent interface is formed between the α-Al matrix and the nanoscale Si precipitates.
(3) The bending strength of the aged SiC/Al-50Si composites prepared at 3 GPa is 387.6 MPa, which is 18.6% higher than that of the unaged composites.

## Figures and Tables

**Table 1:** Lattice parameters and corresponding Si solubility of α-Al matrix in the composites prepared under HP.

*P* (GPa)	*a*_Al_ (nm)	*x*_Si_ (at.%) (Homogeneous)	*x*_Si_ (at.%) (Si-Rich)
1	0.40446	2.6	2.5
2	0.40393	5.6	5.4
3	0.40340	8.7	8.1

**Table 2:** Bending strength of Al–50Si matrix alloy with different elements or reinforcements.

Matrix (wt.%)	Added Elements or Reinforcements	Bending Strength (MPa)	Fabrication Route	References
Al-50Si		287.9	Hot pressing + Aging	[34]
Al-50Si	0.3 wt.% Sc	331	Hot pressing	[35]
Al-50Si	0.5 wt.% Sc	309.6	Hot pressing	[36]
Al-50Si	0.5 wt.% La	321.5	Hot pressing	[36]
Al-50Si	0.5 wt.% Nb	337.2	Hot pressing	[36]
Al-50Si	1 wt.% Mg	405.1	Hot pressing + Aging	[34]
Al-50Si	20 vol.% SiC	387.6	HP + Aging	This work

## Data Availability

All data and models during the study appear in the submitted article.
